# Decentralising NCD management in rural southern Africa: evaluation of a pilot implementation study

**DOI:** 10.1186/s12889-019-7994-4

**Published:** 2020-01-13

**Authors:** Ashley Sharp, Nick Riches, Annastesia Mims, Sweetness Ntshalintshali, David McConalogue, Paul Southworth, Callum Pierce, Philip Daniels, Muhindo Kalungero, Futhi Ndzinisa, Ekta Elston, Valephi Okello, John Walley

**Affiliations:** 1 Good Shepherd Hospital, Siteki, Eswatini; 2 Nuffield Centre for International Health and Development, Leeds Institute of Health Sciences, University of Leeds, Leeds, UK; 3 Institute of Infection and Global Health, University of Liverpool, Liverpool, UK; 4 Clinton Health Access Initiative (CHAI), Mbabane, Eswatini; 5 Ministry of Health, Mbabane, Eswatini

**Keywords:** Non-communicable disease, NCD, Swaziland, Eswatini, Diabetes, Hypertension, Decentralisation, Health service development

## Abstract

**Background:**

The prevalence of non-communicable diseases, and associated morbidity and mortality, is increasing rapidly in low and middle-income countries where health systems often have limited access and lower quality of care. The intervention was to decentralise uncomplicated non-communicable disease (NCD) care from a hospital to nurse practitioners in health centres in a poor rural district in Eswatini, southern Africa. The objective of this study was to assess the feasibility and impact of decentralised care for NCDs within nurse-led clinics in order improve access and inform healthcare planning in Eswatini and similar settings.

**Methods:**

In collaboration with the Eswatini Ministry of Health, we developed and implemented a package of interventions to support nurse-led delivery of care, including: clinical desk-guide for hypertension and diabetes, training modules, treatment cards and registries and patient leaflets. Ten community clinics in the Lubombo Region of Eswatini were randomly selected to be trained to deliver NCD care for a period of 18 months. Observational data on follow-up rates, blood pressure (BP), glucose etc. were recorded and evaluated. We compared blood pressure and blood glucose measurements between the first and fourth visits and fitted a linear mixed effects model.

**Results:**

One thousand one hundred twenty-five patients were recruited to the study. Of these patients, 573 attended for at least 4 appointments. There was a significant reduction in mean BP among hypertensive patients after four visits of 9.9 mmHg systolic and 4.7 mmHg diastolic (p = 0.01), and a non-significant reduction in fasting blood glucose among diabetic patients of 1.2 mmol/l (p = 0.2). Key components of NCD care were completed consistently by nurses throughout the intervention period, including a trend towards patients progressing from monotherapy to dual therapy in accordance with prescribing guidelines.

**Conclusions:**

The findings suggest that management of diabetes and hypertension care in a rural district setting can be safely delivered by nurses in community clinics according to a shared care protocol. Improved access is likely to lead to improved patient compliance with treatment.

## Background

Non-Communicable Diseases (NCDs) are responsible for 40.5 million deaths globally each year, equivalent to 71% of all deaths [1]. Rates of new NCD diagnoses are rising fastest in low and middle income countries, with these countries experiencing 85% of all ‘premature’ deaths due to NCDs (between the age of 30 and 69) [2]. NCD care is among the tracer interventions used to measure coverage of essential health services under Sustainable Development Goal Target 3.8 ‘Achieve universal health coverage’ [3].

Eswatini (formerly Swaziland) is a country with a population of 1.1 million people in Southern Africa [4]. Similar to many neighbouring countries it was severely affected by HIV/AIDS. Well-coordinated HIV programmes have led to significant reductions in HIV incidence and mortality. At the same time, prevalence of NCDs and lifestyle risk factors have been increasing: The most recent WHO STEPS survey reported rates of obesity to be 31% in women and 8.8% in men [5]. High fasting blood glucose (> 7.0 mmol/l) was found in 17% of women and 11% of men and 22% of adults have hypertension [5, 6]. NCDs are estimated to now be responsible for 37% of all deaths in the country [6].

Traditionally, NCD care in Eswatini has been provided using a centralised model, with the majority of care provided by hospitals. Patients have limited access and commonly present late with symptoms of complications. Hospital services are working at full capacity, with little room for expansion. With increasing burden on hospital resources caused by HIV and now the ‘double burden’ of NCDs, this model of care is no longer sustainable. Neighbouring South Africa has worked to control NCDs through health systems strengthening [7], moving towards Integrated Chronic Disease Management’ [8], emphasising ‘assisted self-care’ where patients are assisted to become more expert and adjust their diet, insulin or drugs within a limited range agreed with their clinician. This is a long-term relationship of on-going education and support provided by (preferably the same) health worker. In 2013, the Eswatini Ministry of Health’s National NCD programme began developing a national NCD strategy to address morbidity and premature mortality caused by NCDs [9]. One component of the strategy has been the development and implementation of a comprehensive integrated NCD service in health centres (called ‘community clinics’ in southern Africa).

The study was conducted within the Lubombo region of Eswatini, a recently drought-stricken area with a high rate of extreme poverty. Lubombo is a predominantly rural region with a population of 212,500 and one of the highest prevalence rates of HIV in the world with 29% of those aged 15 and over infected with HIV [10]. The region has one district general hospital, two health centres and a network of 40 nurse-run primary care community clinics. Good Shepherd Hospital (GSH) is a former mission ‘regional’ (rural district) hospital with primary responsibility for tertiary care in the Lubombo region, and so was the focal point for the study. At GSH in 2011, 1588 outpatient appointments were due to diabetes (DM), 3015 due to hypertension (HT), and 1444 due to cardiovascular disease (CVD); the numbers of patients treated are not known (unpublished data).

Currently GSH has one clinic specifically for diabetes/hypertension, run by a team of nurses and assistants. The community clinics provide hypertensive and diabetic services on an ad hoc basis. Some community clinics provide a refill service for these medications, though supplies are inconsistent and access for patients to NCD medication is unreliable. A few community clinics have a doctor visit once a month to attend to patients for diagnoses and complications. Prior to the study, community clinic staff had not received specific training/ guidance on the management of NCDs. GSH is currently responsible for supervision and support of 18 clinics in Lubombo. This pilot project and prospective cohort study was designed to decentralise NCD care from a regional hospital to nurse-led community clinics in the rural Lubombo region. The objective of this study is to assess the feasibility and impact of decentralised care for NCDs within nurse-led clinics in the Lubombo region, Eswatini, in order improve access and inform healthcare planning in Eswatini and similar settings.

## Methods

This observational study began with preparatory work to ensure the clinics were equipped with necessary equipment including a weight scale, BP machine and glucometer to diagnose and treat type II diabetes and hypertension, along with patient information leaflets. We developed a treatment guideline in the form of a desk guide for cardiovascular disease, diabetes and hypertension, which dealt with: primary prevention, identification and screening, diagnosis, education, treatment and referral. The desk guide describes different thresholds for blood pressure control for patients with only hypertension (140/90 mmHg) compared to patients who also have diabetes (130/80 mmHg). The desk guide states a target fasting blood glucose of 7 mmol/l for effective glycaemic control. We developed and administered a 3.5-day skill-based and interactive, training programme for clinic nurses, which covered all elements of the desk guide including data collection, and was based around a combination of lectures, demonstrations and interactive case study based clinical role-plays. The Chief Pharmacist and national pharmaceutical dispensary made provisions to allow distribution of anti-hypertensive and anti-diabetic medications by trained nurse-practitioners. Clinics were provided with the following data collection tools: patient-held heath record card, client-held health record card, enrolment register, and annual review card.

The patients enrolled in the study were recruited from community clinics based on their health status; being diagnosed with Type II diabetes mellitus (DM), hypertension (HTN), or cardiovascular disease (CVD) in accordance with the International Classification of Diseases criteria. Patients with Type I or Type II diabetes requiring insulin were excluded from the study.

The following information was collected at baseline and during each follow up visit as appropriate: demographics (age, date of birth, sex, occupation, and education level), weight, waist circumference, blood pressure (BP) readings, random/fasting blood glucose readings (RBG and FBG respectively), HIV and TB status, medicines prescribed, and any other conditions the patient may have.

We described the baseline characteristics and the trend in health indicators over time, specifically: mean and standard deviation (sd) of systolic and diastolic blood pressure, the proportion of blood pressure readings above diagnostic and safety thresholds, and fasting blood glucose, and completion of routine clinical tasks. We compared blood pressure and blood glucose measurements between the first and fourth visits and fitted a linear mixed effects model with the difference in magnitude between the first and fourth visit as the outcome (dependent) variable and a random intercept for the clinic, to assess whether any change was significant, while accounting for the clustered nature of the data. We used the Kenward-Roger approximation to get approximate degrees of freedom for the t-distribution to get *p*-values and calculated confidence intervals via the profile likelihood method.

### Ethics approval and consent to participate

Verbal consent for inclusion in the study was obtained from participants on enrolment. As an operational research study, the project was focused on the service outcomes and it was not proposed that written consent be requested from individual patients to secure their participation. This study was approved by the Leeds University Faculty of Medicine and Health Research Ethics Committee and the Eswatini National Health Research Review Board.

## Results

A defined six-month period for enrolment allowed for recruitment of sufficient numbers and the establishment of decentralisation at intervention clinics. Data collection was complete 12 months after enrolment. The total study duration was therefore 18 months (6 months recruitment + 12 months’ follow-up). One thousand one hundred twenty-five patients were recruited to the intervention clinics in total 923 (82%) had hypertension alone, 68 (6%) had diabetes alone and 134 (12%) had both diabetes and hypertension (Table 1). 78% of those recruited to the intervention were women. The mean age for women patients was 61 years and 63 years for men. The average weight of recruited patients with hypertension was 77 kg, compared to patients with diabetes who on average weighed 82 kg. Average waist circumference was 99 cm for hypertensive patients and 102 cm for diabetic patients. Numbers recruited per clinic ranged from 52 to 304. Data were collected for 4437 individual clinic appointments. Problems with data collection and patient losses reduced the number of recorded follow-up appointments, but data was recorded for 573 patients who attended at least 4 appointments.

**Table 1:** Recruited participants by age, sex and diagnosis

	N	%
Age group		
20–30	6	1
30–40	48	4
40–50	143	13
50–60	245	22
60–70	292	26
70–80	189	17
80–90	40	4
90–100	7	1
Unknown	155	14
Gender		
Female	875	78
Male	247	22
Unknown	3	0
Education		
None	419	37
Primary	333	30
Secondary	127	11
Unknown	246	22
Diagnosis		
Hypertension	923	82
Diabetes	68	6
Hypertension & Diabetes	134	12
New patients		
New	49	4
Existing	847	75
Unknown	229	20
Total	1125	100

Data on all other variables is taken from the fixed cohort of 573 patients who attended at least 4 follow-up visits, to allow meaningful comparisons to be made. This group consisted of 43 who had diabetes alone, 79 with diabetes and hypertension and 451 with hypertension alone. We analysed blood pressure separately for patients with and without diabetes due to different target blood pressure, while we analysed fasting blood glucose for all diabetic patients together. Only 18 of these patients were diagnosed during the study period so they are considered together with existing patients.

### Blood pressure control

#### BP control - hypertension only

Table 2 summarises the trends in blood pressure for patients in this cohort with hypertension only (*n* = 451) seen over four visits.

**Table 2:** Trend in Blood Pressure: Hypertension only (n = 451)

Parameter	Value	Visit 1	Visit 2	Visit 3	Visit 4
Systolic BP (mmHg)	*Valid values*	429	434	419	422
	*Mean (sd.)*	149 (28)	142 (24)	138 (22)	139 (24)
	*Range*	90–259	95–222	90–210	86–240
	*Values > 140 (%)*	57	46	39	39
	*Values > 180 (%)*	13	7	5	7
Diastolic BP (mmHg)	*Valid values*	427	432	413	419
	*Mean (sd.)*	86 (14)	81 (13)	81 (13)	81 (13)
	*Range*	49–134	48–141	50–124	50–123
	*Values > 90 (%)*	29	20	19	18
	*Values > 110 (%)*	4	2	1	1

The mean systolic BP for this cohort on enrollment was 149 mmHg (range 90–259 mmHg). By Visit 4 this had fallen to 139 mmHg (range 88–240 mmHg). Mean diastolic BP was 88 mmHg (range 41–134 mmHg) on enrolment and fell to 81 mmHg (range 50–123 mmHg) by Visit 4. The change in both systolic and diastolic blood pressure in this group was statistically significant (*p* = 0.01) (Table 4). Using the target of 140/90 mmHg, 57% of this cohort had poor systolic blood pressure (SBP) control at baseline and 29.3% had poor diastolic blood pressure (DBP) control. These figures fell to 39 and 18% respectively by Visit 4. The variation in valid values may be due to BP not being taken or not being recorded.

A blood pressure reading of above 180/100 mmHg is listed as a safety threshold in the Desk Guide, above which level patients require urgent review. At baseline, 13% of these patients had SBP values above this level and 4.0% had DBP values above this level. These proportions fell to 7.1 and 1.4% respectively over the course of the intervention.

#### BP control - diabetes and hypertension

The treatment target for diabetic patients with hypertension is 130/80 mmHg. Table 3 summarises blood pressure control for the cohort of patients who were managed for both diabetes and hypertension (*n* = 79) and seen for at least four scheduled visits.

**Table 3:** Trend in Blood Pressure: Hypertension AND diabetes (n = 79)

Parameter	Value	Visit 1	Visit 2	Visit 3	Visit 4
Systolic BP	*Valid values*	77	74	76	77
	*Mean (sd.)*	148 (24)	144 (21)	138 (22)	139 (19)
	*Range (mmHg)*	100–218	100–210	94–188	108–194
	*Values > 130 (%)*	75	72	59	58
	*Values > 180 (%)*	10	7	5	4
Diastolic BP	*Valid values*	75	74	76	77
	*Mean (sd.)*	86 (15)	82 (12)	83 (12)	81 (10)
	*Range*	41–131	52–107	48–120	59–102
	*Values > 80 (%)*	63	51	49	47
	*Values > 110 (%)*	3	0	3	0

Average SBP readings were similar to those seen in the hypertension cohort; ranging from systolic 148 mmHg at Visit 1 (100–218 mmHg) to 139 mmHg at Visit 4 (108–194 mmHg) and diastolic 86 mmHg at visit 1 (41–131 mmHg) to 81 mmHg at visit 4 (59–109). Table 4 shows the results of a linear mixed effects model of the change in BP and blood sugar accounting for clustering by clinic. Note the number of observations refers only to those observations where both the first and fourth visit values were complete. The change in both systolic and diastolic blood pressure in this group was statistically significant (*p* = 0.04). At baseline, the proportion of diabetic patients failing to reach the target threshold for SBP (130 mmHg) was 75%, falling to 58% by Visit 4. The proportion having poor DBP control also improved from 63 to 47%. Only small numbers of diabetic patients exceeded the safety threshold (180/110 mmHg). The overall proportion with SBP greater than 180 fell from 10 to 3.9% between Visit 1 to 4.

**Table 4:** Linear mixed effects models of change in blood pressure and fasting blood glucose between first and fourth visits

Clinical group	Parameter	Difference	Lower 95% CI	Upper 95% CI	*P* value*	N Observations	N groups
Hypertension only	Systolic BP	−9.9	−13.2	−6.0	0.01	405	10
	Diastolic BP	−4.7	−7.1	−2.3	0.01	402	9
Hypertension and diabetes	Systolic BP	−8.2	−14.1	− 1.8	0.04	75	7
	Diastolic BP	−5.4	−9.4	−1.7	0.04	73	7
Fasting blood glucose diabetic patients	Fasting blood glucose	−1.2	−2.7	0.3	0.2	37	7

### Blood glucose control

Fasting blood glucose (FBG) was the diabetic parameter most frequently recorded in this intervention. Table 5 shows the trend in FBG for patients with diabetes (*n* = 122 who attended four visits or more. The mean fasting blood glucose for this cohort on enrollment was 9.6 mmol/l (range 4.2–22 mmol/l). By Visit 4 this had fallen slightly to 8.9 mmol/l (range 4.4–25.3 mmol/l). This change was not statistically significant (*p* = 0.2). There were fewer observations for fasting blood glucose which was not assessed as regularly as blood pressure.

**Table 5:** Trend in fasting blood glucose control (*n* = 122)

Parameter	Value	Visit 1	Visit 2	Visit 3	Visit 4
Fasting blood glucose (mmol/l)	*Valid values*	81	75	70	75
	*Mean (sd.)*	9.6 (4.3)	8.8 (3.8)	9.4 (3.9)	8.9 (4.0)
	*Range*	4.2–22	3.8–23	3.9–23	4.4–25
	*Values > 7 (%)*	62	56	64	60
	*Values > 10 (%)*	40	31	34	31

Fasting blood glucose readings over 10 mmol/l indicate poor control. The proportion of these readings reduced from 40% at baseline to 31% after four visits.

### Medication usage

Studying the defined cohort of 573 patients who attended four or more visits allows patterns in the usage of medication for diabetes and hypertension to be analysed. The Desk Guide states that patients should be started on Metformin and have a Sulfonylurea (typically Glibenclamide) added if control is poor (or if Metformin cannot be tolerated). At baseline, 17 patients (17%) of diabetic patients on medication were on Metformin only, compared to 8.1% on Glibenclamide only and 79% taking both Metformin and Glibenclamide. The numbers on dual therapy increased slightly over the course of the intervention. By visit 4, 83 diabetic patients (82%) were on both Metformin and Glibenclamide.

### Process indicators

Data was also captured on process indicators describing how closely the delivery of the intervention met the standards outlined in the Desk Guide. The Desk Guide advised that patients should attend community clinics for monthly reviews. For each NCD visit nurses were expected to provide health education and to complete tasks including weight and BP checks (all patients) and blood glucose (diabetic patients).

The completion of routine tasks required by the intervention is illustrated in Table 6 (based on the cohort attending at least 4 appointments). Weight and BP were checked for almost all patients throughout the intervention. Blood glucose was measured less reliably, and the proportion fell during the intervention from 75% of diabetic patients at Visit 1 to 68% of patients at Visit 4. At least three-quarters of patients were documented to have received some form of health education at every visit, although this proportion appeared to fall over the intervention. Health education included: diet, sugar, salt, medication adherence, exercise, smoking and stress management.

**Table 6:** Completion of routine clinical tasks over time

Task	Hypertension				Diabetes			
	Visit 1	Visit 2	Visit 3	Visit 4	Visit 1	Visit 2	Visit 3	Visit 4
BP checked	96%	96%	93%	94%	97%	95%	96%	94%
Blood glucose checked	N/A	N/A	N/A	N/A	75%	70%	63%	68%
Weight taken	83%	95%	93%	93%	73%	90%	89%	89%
Health education given	90%	80%	78%	77%	98%	77%	77%	75%

Table 7 shows that over the course of the intervention the average intervals (in days) between appointments remained relatively stable at just over the recommended monthly interval. There was no clear evidence of a drop-off in appointment intervals as the intervention progressed (based on data for all patients).

**Table 7:** Average intervals between appointments

Interval	Median (days)	Interquartile range (days)	n
Appointment 1–2	42	28–104	665
Appointment 2–3	35	28–84	514
Appointment 3–4	40	28–104	436
Appointment 4–5	58	28–185	128
Appointment 5–6	29	28–58	199

## Discussion

A large cohort of patients were found to have improving trends on average with treatment in all physiological parameters (including extreme values) over four visits. A statistically significant reduction in blood pressure was observed between the first and fourth visits among hypertensive patients with and without diabetes, however no significant change in blood glucose was observed and a significant proportion of patients remained above targets at follow-up. BP was checked in nearly all patients at all visits. Glucose was checked at more than 2/3rds of diabetic patient visits (though a lower percentage in visit 3 for unknown reasons). Health education was given to the large majority of patients, especially at the first visit when care was initiated. Medication usage demonstrates that patients’ drug/doses and were ‘stepped-up’ during the period, as recommended by the Desk Guide, with a trend towards the combination regimes. The process indicators demonstrate the ability of nurses in community clinics to successfully deliver the components of NCD care.

This study adds to the evidence base of similar health system interventions in low and middle-income countries. Recent experience in Pakistan developing case management packages played an important role in helping to prioritise diabetes, hypertension, CVD, asthma and chronic obstructive pulmonary disease prevention across Punjab Province, leading to better provincial planning and budgeting and demonstrating that primary and secondary public health facilities can effectively deliver integrated NCD care in a developing country context [11–13]. Those research-informed guides and tools have been scaled up for use across the 150 public hospitals in Punjab Province. In China, it was shown that by implementing a cardiovascular disease (CVD) care package by family doctors in rural township hospitals can improve prescribing practice and encourage modest improvements in lifestyle changes such as quitting smoking and reduced salt and alcohol intake [14, 15]. Additional measures such as innovative patient education and improved health insurance coverage for outpatients could have helped the CVD care package to be even more effective. The CVD/diabetes care package was adopted and scaled up in the control hospitals. These studies along with our example show that implementing an enhanced package of care in an integrated fashion is both feasible and scalable in low and middle-income settings. In Eswatini the package has been adopted nationally, with plans and funding to scale up across the country.

There were several operational challenges that arose and lessons can be learned from these for future service developments. Despite the agreement to allow clinics to order drugs, there were still problems with organising distribution to the community clinics, which incurred long periods of stock outs. There were often issues with transportation, specifically availability of vehicles and accessibility as many community clinics are not located on roads that are well-paved, with greater difficulty after heavy rainfall. Community clinic nurses have many other duties, such as child health, limiting their attention to diabetes and hypertension care. Specialist nurse supervision from the hospital helped to maintain enthusiasm for the intervention among the stakeholders, but it was challenging to sustain these visits at the necessary frequency. Patients with abnormal parameters or poor response to treatment were required by the protocol to be reviewed by a hospital doctor. This was an access a barrier which resulted in some patients remaining on sub-optimal treatment. This barrier could be overcome if, as for HIV and TB services in Eswatini, doctors visit and see complex cases at the community clinics.

### Limitations

The observational nature of the data means that no firm conclusion can be drawn as to whether care under the new model is better or worse than current practice. The change in blood pressure between the first and fourth visits was statistically significant, but regression to the mean cannot be ruled out. There were significant losses to follow up, which may have been due to patients declining further treatment or seeking treatment elsewhere or may have also been an apparent loss due to poor record keeping. There is a risk of bias due to differential loss to follow up, where the least healthy or compliant patients drop out or keep poorer records, meaning the final sample may not be representative of the general population. There was consistent rotation of nursing staff on the local level presenting challenges. The geographical remoteness of the clinics meant central monitoring of these processes was challenging. The style of recording patient information in Eswatini involves medical staff writing signs, symptoms, vital statistics, diagnoses, and prescriptions in a patient-held notebook, which the patient keeps together with any other clinical documents. These patient-held records may have been well utilised, along with the patient-held cards used in the study, however, because these were kept by the patient, it was not possible to include their data. Given the limitations of this study design, further studies would be necessary to provide stronger evidence of benefit, perhaps including a ‘routine care’ control group. That said, the model proved to be feasible and accessibility of care was improved, and these results add to the evidence base supporting the decentralisation of NCDs in similar settings.

## Conclusion

These findings suggest that decentralization of care for NCDs in low resource settings is feasible and associated with improvements in physiological parameters, though continued training and supervision are required to embed change. Nurses in participating community clinics in Lubombo are now better equipped to recognise and correctly diagnose hypertension and diabetes, and they can initiate treatment and follow up uncomplicated patients. They are also able to follow up and monitor more complex cases and provide refills of drugs normally only available in hospital. Finally, they are using a record keeping system that will help with follow-up care and identify loss to follow-up or treatment failure, though the quality of recording has deteriorated, and further support and supervision is needed to improve this.

## Data Availability

The datasets generated and/or analysed during the current study are not publicly available due to this not being specified in the ethics approval obtained by the Eswatini National Health Research Review Board but are available from the corresponding author on reasonable request.

## Abbreviations

AIDS: Acquired immune deficiency syndrome
BP: Blood pressure
CVD: Cardiovascular disease
DBP: Diastolic blood pressure
DM: Diabetes mellitus
FBG: Fasting blood glucose
GSH: Good Shepherd Hospital
HIV: Human immunodeficiency virus
HT: Hypertension
mmol/l: Millimoles per liter
NCD: Non-communicable disease
RBG: Random blood glucose
SBP: Systolic blood pressure
sd: Standard deviation
STEPS: STEPwise approach to noncommunicable disease risk factor surveillance
TB: Tuberculosis
WHO: World Health Organisation
